# Measurements of surface scale changes in different denture base materials by stereophotogrammetric technique

**DOI:** 10.34172/joddd.2021.001

**Published:** 2021-02-13

**Authors:** Gonca Deste Gökay, Pelin Özkan, Rukiye Durkan, Perihan Oyar

**Affiliations:** ^1^Department of Prosthodontics, Faculty of Dentistry, Bursa Uludağ University, Bursa, Turkey; ^2^Department of Prosthodontics, Faculty of Dentistry, Ankara University, Ankara, Turkey; ^3^Department of Prosthodontics, Faculty of Dentistry, Afyonkarahisar Health Sciences University, Afyonkarahisar, Turkey; ^4^Department of Dental Prostheses Technology, Health Services Vocational High School, Hacettepe University, Ankara, Turkey

**Keywords:** Denture bases, Prostheses, Stereophotogrammetry

## Abstract

**Background.** This study aimed to evaluate the surface scale changes in the denture base material using different polymerization techniques, such as heat-cure/pressure polymerization system and injection molding technique with the stereophotogrammetric technique. The function of a complete denture is related to the adaptation of its base to the supporting areas. Proper adaptation of the base depends on the stability and retention of dentures. The surface scale changes of dentures during processing and in service are of great importance since they affect the denture base material’s fit.

**Methods.** This study focused on the use of a computer-assisted stereophotogrammetric method for measuring changes in the volume of three different denture base resins of an edentulous maxillary ridge. A stone master model simulating the shape of an edentulous maxillary arch was used to prepare three groups of denture base resins. The stereophotographs were evaluated to determine the surface scale differences of maxillary jaws.

**Results.** The results showed no significant differences between the denture borders for three denture base materials (*P* > 0.05).

**Conclusion.** In the evaluation made using this technique, no significant difference was found in the different polymerization techniques in terms of surface scale changes for three denture base materials. Stereophotogrammetry, especially the digital stereophotogrammetric technique, has several useful research applications in prosthodontics.

## Introduction

Acrylic resins are generally used in dentistry, especially as orthodontic appliances, denture base materials, denture repair materials, and provisional restorations.^1^ Polymethylmethacrylate has usually been used as a denture base material because it is inexpensive, durable in oral fluids, and easily manipulated and repaired. Polymethylmethacrylate does, however, have some disadvantages.^2-4^ Complete denture involves replacing the missing natural dentition and associated structures of the maxilla and mandible for patients who have lost all their natural teeth.^5^ To be edentulous is a significant handicap, not only from a psychological point of view but also because of the physical stress caused by the adaptation and retention of a denture. The function of a complete denture is related to the adaptation of its base supporting areas. Better adaptation of the denture base provides stabilization and retention.^6^ With poor adaptation, functional instability, patient dissatisfaction, ulcerations of the oral mucosa, and alveolar bone resorption might occur.^7^ Unavoidable dimensional changes of polymethylmethacrylate-based dentures remain a problem for both patients and dentists during polymerization, such as thermal expansion on heating and contraction after polymerization.^8,9^ The dimensional stability of dentures during processing and in service is critical for denture fit and patient satisfaction.^10^ Further developments in denture base materials and polymerization methods have led to the marketing of new types of resins: high-impact resins, rapid-cured acrylics, light-activated resins, and resins specially designed for microwave curing.^11-13^ The dimensional stability of denture base materials has been extensively studied by estimating the changes occurring across the vertical, horizontal, and sagittal planes using various measurement methods.^14-16^ Unfortunately, there is a paucity of data in clinical experimentation and laboratory studies on three-dimensional (3-D) measurements of complete dentures. One optical method in the medical and dental fields, photogrammetry, is becoming popular for determining deformation properties in three dimensions and eliminating manual measurement errors.^17^

Photogrammetry might be defined as the art, science, and technology of obtaining reliable information about physical objects through the processes of recording, measuring, and interpreting photographic images. Photogrammetry can be briefly described as a science in which information and precise measurements are taken through photographs. After the invention of photography in the 1830s, photogrammetry science developed as analog, analytical, and now digital. In analog photogrammetry, photographs obtained by classical stereoscopic methods are evaluated through optical, mechanical, and optical-mechanical instruments. In analytical photogrammetry, the photographs obtained from the same stereoscopic methods are evaluated using instruments supported by a computer. In digital photogrammetry, photographs are recorded in hard copy or electronically obtained CCD digital cameras and evaluated with computer techniques that simulate human eyes and recognition, and the resulting products are obtained.^18^ Medical photogrammetry is the term used to cover all photogrammetry applications within the broad field of medicine. These applications are numerous, but the majority relate to measuring the shape and size of body parts and changes in these parts’ form over time.^19^ The primary tool in medical photogrammetry is stereophotogrammetry, but other forms of imagery, such as moiré patterns, fringe interferometry, and x-ray, are also used. Although primarily applied to cartography, photogrammetric skills and techniques are used in various dental metrological problems. Many applications have been reported in these fields, and the majority relate to the determination of the volume and surface areas of the whole body or various body parts, such as the trunk and limbs, head, teeth, and jaws. Direct measurement of a part of the head is a difficult task. The face is easily distorted by pressure on the soft tissues.

Furthermore, the general shape is complex, and some areas, particularly around the eyes, are very sensitive to touch. Photogrammetry overcomes many of these difficulties and can provide an alternative means of measurement; therefore, dentists and, in particular, orthodontists have shown great interest in using photogrammetry to obtain measurements of changes in shape resulting from growth, surgery, or dental treatment.^20^ All the photogrammetric systems used in dental research employ the most frequently used type of photogrammetry, the close-range type, in which the object-to-camera distance is limited. Some advocate 300 mm as a maximum limit, while the minimum distance is a fraction of a millimeter when macro- and microscopic photographs are used for measurements.^21^ The null hypothesis was that different polymerization techniques would change the surface scale of three denture base materials.

## Methods

For this study, a stone master model was prepared to simulate the shape of the edentulous upper arch. The maxillary arch was studied because of the relative ease with which topographic landmarks could be identified, marked, and located on the stereoscopic photographs. Three edentulous maxillary models were obtained from this stone master cast. The base plate and cast were adapted to the stone models. Because it measured the inner surface of dentures, the artificial teeth were not placed on the model. Complete dentures were finished using different acrylic denture base resins.

### Materials and specimen preparation

**Figure 1:**
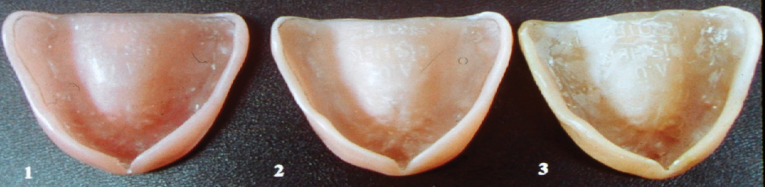
Acrylic prostheses (1. QC-20, 2. SR-Ivocap, 3. Dental D).

**Figure 2:**
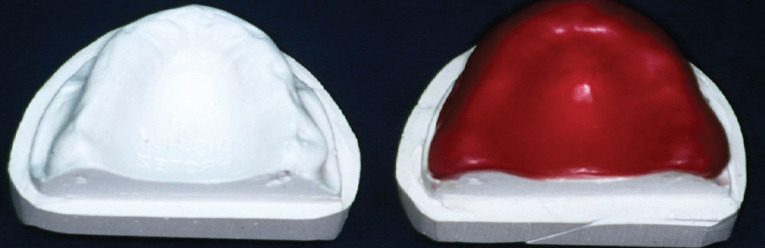
Stone master model simulating the shape of edentulous maxillary arch.

In the first group, polymethylmethacrylate-containing conventional acrylic resin (QC-20, Dentsply, England) was used and polymerized with a heat-curing/pressure system. High-impact acrylic resin (SR-Ivocap, Ivoclar, Liechtenstein) was preferred and polymerized by an injection molding technique for the second group. This system is a specially developed injection technique that compensates for curing shrinkage. For the last group, an acetal resin and thermo-injection system were used (Dental D, Quattroti, Italy). Dental D is a thermoplastic polymer (polyoxymethylene) composed of solid tablets placed in a cartridge and processed in a special MG-Newpress oven. The waxed case is injected into the Dental D flask, the wax is eliminated by boiling it out, and the case is processed with the MG-Newpress injection system. Dental D thermoinjection occurs by inserting the cartridge containing the selected shade into the MG-Newpress, which has been pre-heated to 220°C. After 20 minutes, the pre-heated and boiled-out flask is positioned in the MG-Newpress and injected. After injection, the MG-Newpress is turned off, and the Dental D material cools down for 30 minutes. The difference is the cold-cured injection of common acrylic resins for pressing. The acrylic resin prostheses and stone master models were prepared according to the manufacturer’s instructions (Figures 1 and 2). The denture base resins used in this study are presented in Table 1.

**Table 1:** Denture base materials used in this study

**Denture base resin**	**Manufacturer**	**Type**	**Polymerization method**
QC-20	Dentsply, England	Polymethylmethacrylate	Heat curing, conventional resin
SR-Ivocap	Ivoclar, Liechtenstein	Polymethylmethacrylate	Injection molding, heat curing
Dental D	Quattroti, Italy	Polyoxymethylene	Thermo injection, heat curing

### Stereophotogrammetric analysis

**Figure 3:**
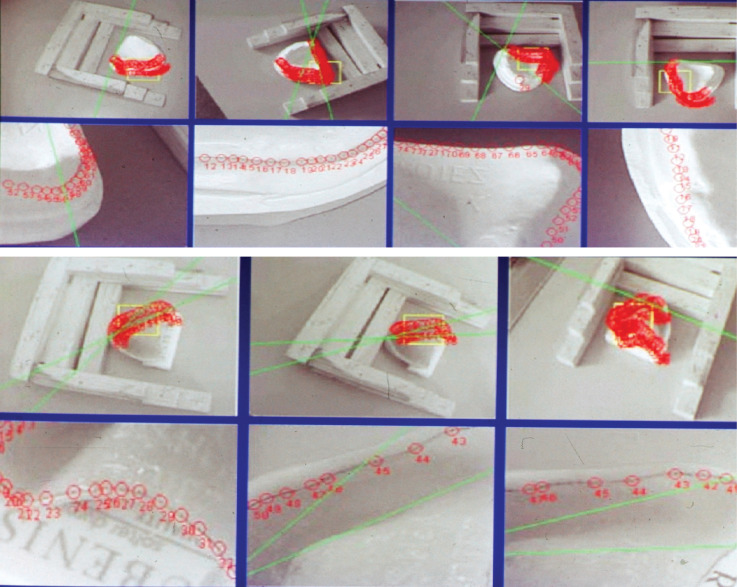
Photographs were taken with Nikon Coolpix P950 Digital Camera for stereophotogrammetric analysis.

After the laboratory stage, photographs were taken with a Nikon Coolpix P950 digital camera for stereophotogrammetric analysis (Figure 3).

Some technical information of this camera is as follows:

- Resolution of the sensor: 2.1 million pixel CCD
- Output resolution: 1600×1200 pixels
- Disc capacity: 96 MB
- Focal length: 7–21 mm
- Exposure interval: 1/25–1/500 seconds
- Diagram: 1:26–4

All the photographs were taken as stereoscopic in the laboratory, and the model and the dentures were lit during the exposure. For the digital photogrammetric restitution, PICTRAN software, developed by Berlin TU, was employed in this study. The smallest element of a digital image or digitized photograph hard copy is a pixel. Pixels have a value of 0 to 255 on the gray scale. On the photographs, 4–6 different images exist in each image series (red-green-blue).

This photogrammetry system takes 10 pictures per second with a margin of error of under 10 μm between two control points. The number of photographs and control points can be seen in Table 2.

**Table 2:** Number of photographs and control points and mean values and standart deviations of data obtained from the restitution of photographs

**Materials-techniques**	**Number of photographs**	**Number of control points**	**Measurements of dentures’ inner surface (mm** ^2^ **)**	**Measurements of denture borders (mm)**
Master model	4	16	2029.02 ± 37.24	172.786 ± 14.23
QC-20 Conventional resin heat-cure/press	5	14	2060.70 ± 29.41	173.550 ± 15.32
SR-Ivocap high-impact resin injection molding	5	15	2123.43 ± 31.25*	175.441 ± 16.89
Dental D thermoinjection	4	7	1936.27 ± 26.81*	170.207 ± 14.77

*Means are statistically different at *P*= 0.05.

The X and Y coordinates of the control points were evaluated with Jena coordinatograph instruments in terms of mp_xy_ = ±10-micron accuracy. The Z coordinates of the control points were evaluated with mechanical dividers at mz = ±100-micron accuracy.

*Interior orientation elements*:

F: 7.0601400 mm

X_h_: -0.0113 mm

Y_h_: -0.006605 mm

Interior orientation using fiducial marks, the measurement of object points, and exterior orientation were made with PICTRAN B modules.

In the evaluation of digital photogrammetry, a mathematical equation can be described to transform the photographs vertically.

x`` = (a* x` + b* y` + C) / (g* x` + h* y` + 1)

y`` = (d* x` + e* y` + f) / (g* x` + h* y` + 1)

*x``, y`` = coordinates of two-dimensional object*

*x` y` = coordinates of measured photographs*

*a, b, c, d, e, f, g, h = unknown parameters*

The statistical data obtained from the restitution of photographs are presented in Table 2. The data were analyzed using the repeated-measures ANOVA (*P*< 0.05).

## Results

The null hypothesis was rejected because the results showed no significant differences between the denture borders between the three denture base materials and polymerization techniques. Measurement of inner surfaces showed that Dental D was significantly different from the other two denture base resins. Photogrammetric denture area measurements revealed that the Dental D denture was significantly better than the other two dentures. It exhibited minimal surface scale changes after polymerization. The denture fabricated with the injection molding technique, SR-Ivocap, had the maximum surface scale changes compared to the other two denture base materials. The denture fabricated with injection molding technique had the largest area than the master model and the other dentures.

Dental D, the thermo-injection system, exhibited lower surface measurements than the master model, both in inner surfaces and denture borders.

## Discussion

Stereophotogrammetry is mainly concerned with reconstructing three-dimensional objects from a pair of overlapping two-dimensional perspective views of the objects. Each perspective’s view is usually a photograph that records the focused image of the object. Photogrammetric measurement techniques make it possible to adopt common standards for data and image archiving. The patient data can be easily compressed, transferred, and encoded. Many reports have been published on the use of photogrammetry in facial studies. As early as 1992, Mansbach^22^ demonstrated that stereophotogrammetry was of potential use in the study of orthodontic casts. Zeller^23^ used this technique in an in vitro study to evaluate the contour of the face and tooth restorations.The face has been the subject of studies by many researchers, among them, Bjorn et al^24^ followed erupted and unerupted lower molars, and Burke and Beard^25^ monitored the growth of facial soft tissues.

Coope et al^26^ and Dixon and Newton^27^ used a system similar to that of Burke and Beard^25^ to demonstrate minor clinical abnormalities, cherubism, and minimal form of the cleft syndrome. In Ucok’s research, postoperative edema formation was registered by stereophotogrammetry after third molar surgery.^28^ Motta et al^29^ used photogrammetry to assess the relationship between head posture and the incidence of bruxism in 42 children. Kau et al^30^ evaluated soft tissue changes during dentofacial growth and changes in the mandibular growth pattern after mandibular segmental resection. Craniofacial photographic analysis has also been used by Lee et al^31^ to predict obstructive sleep apnea. In restorative dentistry, Zulqar-Nain et al^32^ developed a system used for orthodontic brackets on the buccal surfaces of the lower first premolars. Browning et al^33^ used this technique to measure the movement of partial denture-clasp assemblies and associated tooth abutments. Özkan and Aydın^34^ investigated the deformation properties of Kennedy Class I removable partial dentures by stereophotogrammetry. The system described by Adams et al^35^ was used in the stereophotogrammetric analysis of certain dental features associated with dental restorative procedures. Many clinical reports have described the use of photogrammetry to record the positions of multiple dental implants to prepare immediate multiple implant-supported fixed provisional restorations.^36-39^

Chadwick et al^40^ used this technique to quantitatively assess the wear of dental restorative materials. Adams and Wilding^41^ used biostereometric techniques to record both short- and long-term changes in residual alveolar ridge morphology. A digital photogrammetric technique was used in another study measuring the relative dimensional changes before and after rapid maxillary expansion. The transverse diameters and volumetric variations of the palate were measured by photogrammetry on study casts taken at three different phases of therapy.^42^ Many studies have used different techniques to examine the dimensional changes of the denture base resin.

Consani et al^43^ studied the effect of simulated microwave disinfection on maxillary denture base adaptation using two different flask-closure methods. Three transverse cuts were made through each stone cast-resin base set, corresponding to the distal aspect of the canines, mesial aspect of the first molars, and posterior aspect of the palatal region. Measurements were made in the bases using an optical micrometer at 5 points for each cut to determine adaptation: left and right marginal limits of the flanges, left and right ridge crests, and midline. Moturi et al^44^ used a computer imaging system to compare dimensional changes in polymethylmethacrylate complete denture bases resulting from three different cooling regimens following a standard heating cycle. Duymuş et al^45^ used a traveling microscope to measure the total distance from the uppermost dimple to the lowest dimple to the acrylic resin blocks. Arafa^46^ used digital calipers to assess the dimensional stability, including thermal changes, of three different denture base materials.

Savirmath and Mishra^47^ measured linear dimensional changes in three pre-determined points on the specimens of all the groups using a traveling microscope after removing the sample from the flask. Savabi et al^48^ measured linear dimensional changes in the anteroposterior and mediolateral distances and vertical changes in the first molar region. In the present study, computer-assisted stereophotogrammetric methods were used to measure changes in the volume of three different denture base resins. In this in vitro study, there was no significant difference between the three denture base materials for denture borders. Photogrammetric denture area measurements revealed that denture fabricated with the Dental D thermo-injection system was significantly better than the other two dentures. It exhibited minimal surface scale changes compared to the other resins. It also had less surface area than the master model. Its processing technique might have caused these differences because of more contraction after polymerization. The denture fabricated with the SR-Ivocap injection molding technique had the largest area among the master models and the other dentures. Different processing techniques might have caused these different surface scale changes.

The limitation of the present study was that a small number of acrylic resin materials were used. Surface scale changes of acrylic resins can be investigated by performing different polymerization methods and measurement techniques or can be compared using digital stereophotogrammetric technique and other measurement techniques. In addition, it is necessary to substantiate the results of this study by further clinical studies.

## Conclusion

Stereophotogrammetry, especially the digital stereophotogrammetric technique, has several useful research applications in prosthodontics. Within the limitations of this in vitro study, the following conclusions were drawn:

1. Stereophotogrammetry can be used to quantitatively evaluate edentulous arch forms, residual ridge resorption, and the fit of the denture base, cast, and the denture area.
2. Stereophotogrammetry can show the two-dimensional (X and Y coordinates) and three-dimensional (Z axis heights) qualities of the edentulous arch.
3. Photogrammetric measurements indicated that the area of denture fabricated using high-impact resin had the largest area and that the Dental D denture area was similar to the area of the master model. No significant differences were found between the measurements of the denture border for three materials and techniques.
4. According to the results, Dental D resin can be used to fabricate removable prostheses as an alternative to conventional acrylic resin.

## Authors’ Contributions

GDG and PO contributed in developing the concept of study, preparation of the manuscript and supervision of the study. RD contributed in Proof reading and editing of the manuscript and PO Performed the observations in the study and preparation of the manuscript.

## Funding

No funding.

## Competing Interests

The authors declare that they have no conflict of interest.

## Ethics Approval

Not applicable.
